# Local Substrate Heterogeneity Influences Electrochemical Activity of TEM Grid-Supported Battery Particles

**DOI:** 10.3389/fchem.2021.651248

**Published:** 2021-03-19

**Authors:** Christina Cashen, R. Colby Evans, Zach N. Nilsson, Justin B. Sambur

**Affiliations:** Department of Chemistry, Colorado State University, Fort Collins, CO, United States

**Keywords:** single particle electrochemistry, correlated imaging, ion insertion dynamics, electrochromism, optically detected electrochemistry

## Abstract

Understanding how particle size and morphology influence ion insertion dynamics is critical for a wide range of electrochemical applications including energy storage and electrochromic smart windows. One strategy to reveal such structure–property relationships is to perform *ex situ* transmission electron microscopy (TEM) of nanoparticles that have been cycled on TEM grid electrodes. One drawback of this approach is that images of some particles are correlated with the electrochemical response of the entire TEM grid electrode. The lack of one-to-one electrochemical-to-structural information complicates interpretation of genuine structure/property relationships. Developing high-throughput *ex situ* single particle-level analytical techniques that effectively link electrochemical behavior with structural properties could accelerate the discovery of critical structure-property relationships. Here, using Li-ion insertion in WO_3_ nanorods as a model system, we demonstrate a correlated optically-detected electrochemistry and TEM technique that measures electrochemical behavior of via many particles simultaneously without having to make electrical contacts to single particles on the TEM grid. This correlated optical-TEM approach can link particle structure with electrochemical behavior at the single particle-level. Our measurements revealed significant electrochemical activity heterogeneity among particles. Single particle activity correlated with distinct local mechanical or electrical properties of the amorphous carbon film of the TEM grid, leading to active and inactive particles. The results are significant for correlated electrochemical/TEM imaging studies that aim to reveal structure-property relationships using single particle-level imaging and ensemble-level electrochemistry.

## Introduction

Nanoscale materials are attractive ion insertion hosts for applications such as electrochemical energy conversion and electrochromic smart windows (Bourderau et al., 1999; Li et al., 1999; Graetz et al., 2003; Arico et al., 2005; Manthiram et al., 2008). Their small dimensions minimize charge and ion transport distances, facilitating rapid and reversible charge injection and extraction. However, individual nanoparticles in a sample batch vary in size, shape, and surface structural sites. Understanding how variations among particles contribute to ion insertion dynamics is critical to the design and optimization of electrodes.

Toward this goal, single particle-level electrochemical methods have been applied to battery materials (Heubner et al., 2020). Single particle-level electrochemical measurements reveal underlying ion and electron transport processes that are fundamentally related to solid state chemistry and the solid/electrolyte interface (Nelson et al., 2017; Wolf et al., 2017; Li et al., 2018; Yu et al., 2018). Scanning probe electrochemical methods have been used to uncover heterogeneous electrochemical activity of LiFePO_4_ and LiMn_2_O_4_ battery particles (Kumatani et al., 2014; Tao et al., 2019). Similarly, Tao and co-workers pioneered a widefield plasmonic-based imaging method that has been used to distinguish Li-ion insertion/extraction behavior among LiCoO_2_ nanoaprticles (Jiang et al., 2016). Several groups have attached battery particles to single nano- or micro-electrodes, extracting critical solid state diffusion and interfacial charge transfer rate constants (Jebaraj and Scherson, 2012; Tsai et al., 2018). All the above approaches possess advantages and disadvantages with respect to spatial/temporal resolution and throughput (Heubner et al., 2020). A major disadvantage of the *ex situ* approach is that it removes any possibility of correlating real-time electrochemical and structural dynamics. Another major challenge is that the aforementioned single particle-level methods typically correlate electrochemical and composition/structure information using *ex situ* scanning electron microscopy (SEM). Imaging may be performed before and after single particle-level electrochemical measurements, effectively linking single particle-level electrochemical and structural information. However, the limited spatial resolution of SEM imaging does not permit discovery of atomic scale structure–property relationships.

On the other hand, several atomic-level imaging methods have been used to study ion insertion kinetics of nanoparticle film or slurry electrodes (De Marco and Veder, 2010; Harks et al., 2015; Grey and Tarascon, 2017; Yuan et al., 2017; Tripathi et al., 2018; Boebinger et al., 2020; Li et al., 2020). Transmission electron microscopy (TEM; Huang et al., 2010; Liu and Huang, 2011; Liu et al., 2012; Zeng et al., 2014, 2020; Qi et al., 2016; Xie et al., 2017) and X-ray (Totir et al., 1997; Ota et al., 2003; Deb et al., 2004; Kim and Chung, 2004; Chao et al., 2010; Shearing et al., 2011; Nelson et al., 2012, 2017; Li et al., 2014, 2018; Shapiro et al., 2014; Wolf et al., 2017; Yau et al., 2017; Yu et al., 2018) imaging methods have measured the real-time lithiation kinetics of nanoparticle electrodes and have successfully incorporated material properties such as film porosity, particle shape, orientation, and composition to predict the system's electrochemical response (Garcia et al., 2005; Gupta et al., 2011; Stephenson et al., 2011; Ebner et al., 2014; Landesfeind et al., 2018). *In situ* single particle-level TEM measurements have revealed dynamic structural information of battery materials (Huang et al., 2010). However, the *in situ* TEM technique requires vacuum or quasi vacuum operation conditions and the *in situ* sample holder and sample preparations steps are complicated, leading to low-throughput (Wu and Liu, 2018).

Expanding the scope of high-throughput *ex situ* single particle-level analytical techniques that effectively link electrochemical behavior with structural properties could help uncover important design principles for battery and related functional materials. One strategy that has been applied to nanofibers (Zhang et al., 2018), electrocatalysts (Yu et al., 2012; Arán-Ais et al., 2015), and battery materials (Wang et al., 1999; Lee et al., 2019) is to measure the electrochemical response of the entire electrode and link that response to structural changes of single particles revealed by TEM imaging. Tracking the same particles over time can reveal how electrochemical cycling induces structural transformations. The electrochemical behavior of single battery particles may be inferred from the ensemble-level electrochemical response (Zhang et al., 2020). Inferring single particle-level electrochemical responses from the ensemble-average system response assumes some level of homogeneous activity across the population. One issue is that the TEM grid substrate material has different electrical and mechanical properties than more common metal substrates (e.g., Cu and Ti foil). Hence, understanding the role of the TEM grid support on the electrochemical behavior of particles is important for interpreting *ex situ* TEM results with ensemble-level electrochemical data.

Here, we introduce an optically-detected electrochemical approach that uses a conventional bright field optical microscope to acquire single particle electrochemical data and *ex situ* TEM characterization of single nanoparticles in a one-to-one fashion. The measurements relate the optical density (OD) change of single nanoparticles to redox changes in the particle. We demonstrate the method using Li-ion insertion in tungsten oxide nanorods (NRs) as a model system. The optical signal is insensitive to electrical double layer charging but sensitive to redox changes of elements in the particle (e.g., W^6+^/W^5+^ in WO_3_). Widefield optical imaging has been used to characterize Li-ion insertion in electrochromic WO_3_ and MoO_3_ thin films (McEvoy and Stevenson, 2003, 2005a,b; Kondrachova et al., 2009) and battery materials at the ensemble- and single microparticle-levels (Harris et al., 2010; Love et al., 2015; Duay et al., 2016; Wood et al., 2016; Sanchez et al., 2020), but the aforementioned studies have not linked electrochemical and composition/structural analysis at the single particle-levels. This widefield approach enables one-to-one electrochemical-to-structural characterization of tens to hundreds of particles in a single experiment, limited by the coverage of single particles on the TEM substrate.

## Experimental Methods

### Synthesis and Characterization

Hexagonal WO_3_ (h-WO_3_) NRs were synthesized via a hydrothermal reaction that was developed by Wang et al. (2008). We added 0.5798 g of NaCl (Fisher Scientific) and 0.8250 g of Na_2_WO_4_ ∙ 2H_2_O (Mallinckrodt Chemical Works) to 19 ml of 18.2 MΩ-cm H_2_O. Then, under continuous stirring, 3 M HCl was slowly added to adjust the solution pH to 2.06. This procedure typically required 850 μL of 3 M HCl. The pH was monitored by a HQ11d pH probe (Hach). This solution was then placed into a 23 ml Parr acid digest reactor and heated in a convection oven at 180°C for 24 h. After 24 h, the vessel was removed from the oven, placed on the bench top, and allowed to cool to room temperature. The supernatant was pipetted out of the vessel and the white powder product at the bottom of the vessel was transferred to a centrifuge tube, washed with 18.2 MΩ-cm H_2_O, and stored in ethanol. The product was analyzed via powder X-ray diffraction (PXRD) using a Bruker D8 Discover Series II X-ray Diffractometer (PXRD), with Cu Kα (λ = 0.15406 nm) radiation at 50 kV and 50 mA in a 2θ range from 10° to 80° (Supplementary Figure 1). The PXRD experiments were carried out at room temperature on a zero-diffraction silicon wafer (MTI Chemical Corp).

### Electrochemical Cell Assembly

**Figure 1:**
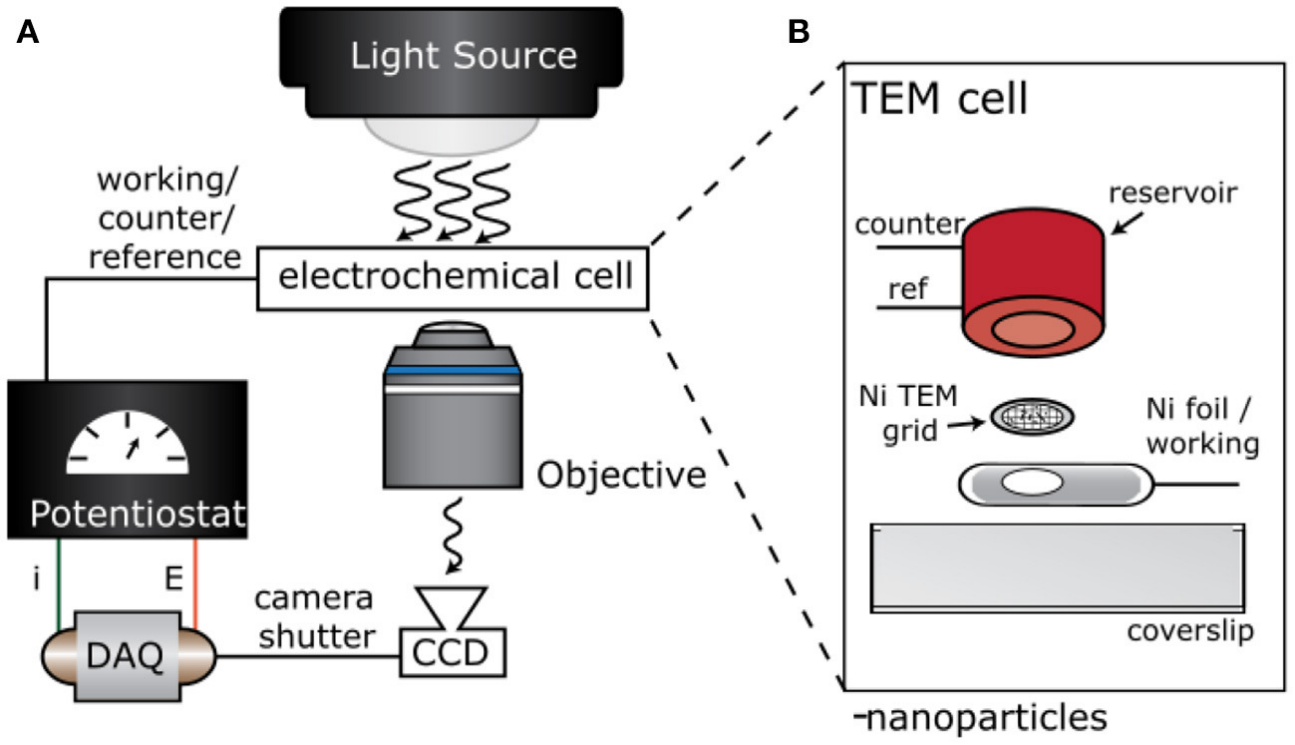
Experimental setup and electrochemical cell design. Cartoon illustration of the **(A)** experimental setup for single particle electro-optical imaging experiments and **(B)** electrochemical cell with a TEM grid working electrode.

Figure 1A shows a cartoon illustration of the experimental setup and electrochemical cell design. We designed an optically transparent electrochemical cell with a TEM grid working electrode. To do so, we drop-casted a 1 mg/ml ethanol solution of WO_3_ nanoparticles on a Ni TEM Grid (Ted Pella PELCO® 200 Mesh Ni Grid, pure carbon). The TEM grid was sandwiched between a 3D printed polyethylene terephthalate glycol (PETG) reservoir and a small piece of Ni foil (Sigma-Aldrich, >99.9% Ni), as shown in Figure 1B. The Pt counter (CE) and Ag/AgCl reference (RE) electrodes were secured in a 3D-printed PETG reservoir via pre-drilled holes and epoxy (Figure 1B). The grid was placed above a 3 mm hole in the Ni foil that was cut with a tap and dye set. Once the TEM grid was sandwiched between the coverslip, Ni foil, and reservoir, the entire assembly was mechanically secured using insulating epoxy (Loctite E-120HP Hysol). A digital multimeter confirmed that the TEM grid and Ni foil remained in good electrical contact throughout the assembly process.

### Electro-Optical Imaging Experiments

The experimental setup and detailed image analysis procedures are provided in our previous publications (Evans et al., 2019a,b). Briefly, the electrode assembly was mounted on the motorized XY stage of an Olympus IX-73 optical microscope. Bright field transmission images were acquired at a 100 ms frame rate and under 940 nm light emitting diode (LED, Thor Labs) excitation. The light was collected by a 100× objective (UPLANSAPO100x/W) and imaged on an Andor (iXON 897) EM-CCD detector. A 2× magnification lens was inserted into the beam path to achieve 200× magnification. Chronoamperometry measurements were conducted in 1 M LiClO_4_ (Aldrich) in propylene carbonate (Aldrich) using a potentiostat (Metrohm Autolab PGSTAT128N). Cathodic and anodic potential steps were applied for 30 s between −1.0 and +0.5 V vs. Ag/AgCl, respectively. The applied potential, electrochemical current, and EM-CCD camera shutter signals were acquired simultaneously via a data acquisition card (DAQ) to precisely synchronize the signals (Evans et al., 2019b). This data synchronization enables each image in the stack or “movie” to be indexed to the time and potential as measured by the potentiostat.

### Electron Microscopy

*Ex situ* electron microscopy experiments were performed after the electro-optical imaging experiments. The TEM grid was mechanically removed from the cell using a razor blade. The grid was gently washed with dimethyl carbonate (Sigma) and dried in a N_2_ stream. Transmission electron microscopy images were collected on a JOEL JEM-2100F electron microscope at a working voltage of 200 keV.

### Image Analysis Procedures

**Figure 2:**
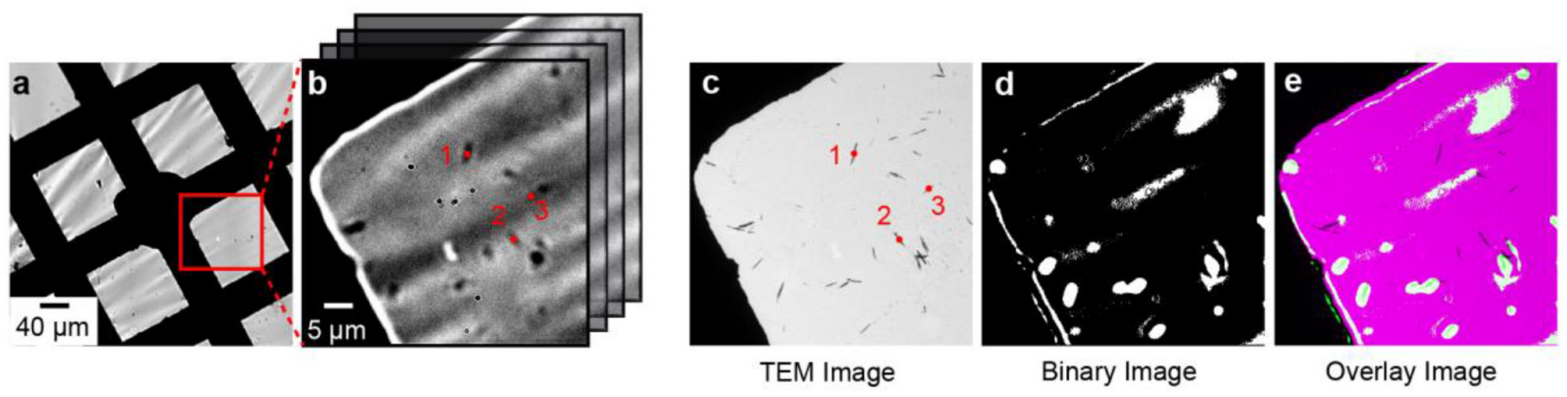
Correlated electro-optical and TEM imaging. **(a,b)** Bright field optical microscope image of a WO_3_-coated TEM grid working electrode at 10× and 60× magnification, respectively. **(b)** Imaging stack showing the continuous imaging sequence in the electro-optical imaging experiment. The centroid positions of single particles are indicated by the red dots labeled 1, 2, and 3. **(c)** TEM image of the sample region in **(b)**. The red dots represent the centroids of individual particles. **(d)** Binary optical image of WO_3_ nanorods. White pixels correspond to OD-values that were >10-times the standard deviation of the background. **(e)** Overlay of the binary image on the TEM image.

We developed an image analysis procedure to overlay the optical and electron microscopy images. The first step of the procedure is to identify control point pairs in both images. For example, the points labeled 1, 2, and 3 in Figure 2b represent the centroid positions of three individual nanorods that were determined using the centroid function in MATLAB's image processing toolbox. Figure 2c shows a TEM image of the same sample region in Figure 2b and the points labeled 1, 2, and 3 represent the centroid positions of the same nanorods. We use the imtransform function in MATLAB to overlay the optical image onto the TEM image using the spatial transformation defined by the control point pairs. Active particles were classified as active if the OD-value exceeded the mean + 3 standard deviations of the background OD.

We developed an intensity-based algorithm to assign particles and particle clusters to specific background region types, as discussed later in the manuscript. We identified four background region types and analyzed intensity vs. time trajectories from each region. We used the mean and standard deviation values of Type 4 areas as criteria for assignment. Single particles and clusters were assigned to Type 1 regions if, following a polarization pulse, the OD intensity surrounding a particle was less than the mean – 3 standard deviations of Type 4 regions. If the particles could not be assigned to Type 1 regions, then they were assigned to Type 3 regions if the standard deviation of the background exceeded 3× the standard deviation of Type 4 areas. If the particles could not be assigned to Type 1 or 3 regions, then they were assigned to Type 4 regions. We omitted the three particles that could be assigned to a Type 2 region because the results were not statistically meaningful.

## Results and Discussion

We used bright field optical microscopy to measure the rate of Li-ion insertion in single WO_3_ nanorods. In a typical experiment, WO_3_ nanorods were drop-casted on the TEM grid and assembled into a 3-electrode electrochemical cell as shown in Figure 1. The average length and width of the nanorods were 1.02 ± 0.54 and 0.10 ± 0.04 μm, respectively, as determined by electron microscopy (Evans et al., 2019a). Figures 2a,b shows 10× and 60× magnification optical transmission images of the WO_3_ nanorod-coated TEM grid working electrode before lithiation. The nanorods appear as dark objects against a bright background because the nanorods absorb and scatter incident light. Figure 2c shows a TEM image of the same sample region as in Figures 2a,b. The amorphous carbon film of the TEM membrane is expectedly transparent in the electron microscope but the material is not fully transparent in the optical microscope, which has important consequences for the electro-optical imaging experiment, as discussed in detail below.

We studied the dynamic optical properties of these electrochromic h-WO_3_ NRs during chronoamperometry experiments. To induce Li-ion insertion in the h-WO_3_ NRs according to Equation (1), a cathodic potential step of −1.0 V vs. Ag/AgCl was applied to the TEM grid electrode while a series of bright field transmission images were acquired at a 100 ms frame rate (Figure 2b). The lithiation reaction increases the OD of the sample due to the formation of [Li^+^-W^5+^] color centers (Bohnke et al., 1992; Vuillemin and Bohnke, 1994), causing the compound to turn dark blue. Applying an anodic potential of +0.5 V induces the delithiation reaction, causing the blue compound to return to a more transparent state (W^6+^).

(1) $$\begin{array}{r} WO_{3}+xLi^{+}+xe^{-}↔{Li_{x}WO}_{3} \end{array}$$

The electro-optical measurements relate OD as a function of polarization time, ΔOD(*t*), to the concentration of [Li-W^5+^] color centers as a function of time, *c*(*t*), using the Beer-Lambert law according to Equation (2) (valid for *x* < 0.5) (Denesuk and Uhlmann, 1996; Scarminio et al., 1999; Wen et al., 2015), where ε_λ_ is the monochromatic molar absorption coefficient (10^6^ cm^2^/mol at 930 nm; Vuillemin and Bohnke, 1994) and *d* is the particle thickness. We measure ΔOD(*t*) by calculating the intensities of the incident and transmitted light through the amorphous carbon film of the TEM grid working electrode and WO_3_ particles, *I*_0_(*t*) and *I*(*t*), respectively, as a function of time. We determine *d* from electron microscopy images and assume that the nanorod cross section can be approximated as a square rectangle, as supported by atomic force microscopy data (Evans et al., 2019a).

(2) $$\begin{array}{l} \Delta OD\left(t\right)=\varepsilon_{\lambda }c\left(t\right)d=-{\text{log}}_{10}\frac{I\left(t\right)}{I_{0}\left(t\right)}+{\text{log}}_{10}\frac{I\left(0\right)}{I_{0}\left(0\right)} \end{array}$$

To identify OD changes of objects during the cathodic polarization pulse, we thresholded the final image by subtracting the initial image (delithiated particles) from the final image at the end of the cathodic potential step (lithiated particles) to form a binary image. Figure 2d shows the result of the thresholding procedure, where white pixels indicate an OD increase resulting from the potential step. Finally, we overlay the binary image in Figure 2d on the TEM image using the image analysis procedure described in the Experimental Methods section. The white pixels located on dark objects in Figure 2e represent single WO_3_ NRs or WO_3_ NR clusters whose OD increases during the cathodic potential pulse and, therefore, those particles that are active for the lithiation reaction.

**Figure 3:**
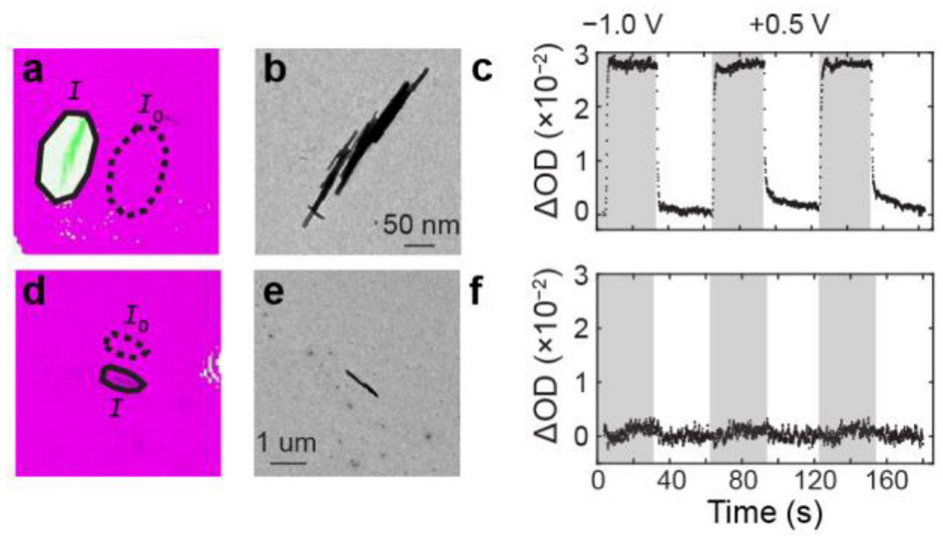
ΔOD(*t*) kinetics of active and inactive WO_3_ particles. **(a)** Optical and TEM overlay image. The solid and dashed ovals represent the *I* and *I*_0_ pixel regions that were used to calculate the OD trajectories. **(b)** TEM image of an active WO_3_ particle cluster. **(c)** ΔOD(*t*) trajectory of the particle cluster in **(a,b)**. The gray and white vertical bars represent the time windows of the cathodic and anodic potential steps. **(d–f)** same as **(a–c)** but for an inactive h-WO_3_ NR.

Having developed an image processing algorithm to assess electrochemical activity of WO_3_ NRs in an unbiased way, we studied the electrochemical activity of 6 single WO_3_ NRs and 83 WO_3_ NR clusters consisting of 2–15 NRs. We identified that 33% of individual NRs and 41% of NR clusters were electrochemically active, as evidenced by OD-values that exceeded the background threshold defined in the Experimental Methods section. Figures 3a–c and Figures 3d–f show representative ΔOD(*t*) trajectories from active and inactive WO_3_ particles, respectively. Upon applying the cathodic potential step of −1.0 V vs. Ag/AgCl, one WO_3_ particle cluster exhibits an OD increase (Figures 3a–c) whereas another WO_3_ particle (Figures 3d–f) exhibits no clear OD change. In our previous study of the same WO_3_ NR sample on indium doped tin oxide (ITO) electrodes, we observed only 9 out of 102, or 9%, of these h-WO_3_ NRs were inactive (Evans et al., 2019a). In addition, we observed that 349 clusters contained 1–25 NRs were electrochemically active. The high percentage of active clusters on ITO substrates is likely due to the fact that the probability of forming a cluster from two inactive single particles is only 0.8%. The particle–particle interfaces and contact area between particles in clusters influence OD dynamics, not overall activity (Evans et al., 2019a). Since the electrolyte and WO_3_ materials in those measurements were identical to those used in this work, we attribute the large inactive particle population in this study to a poor electrical contact between the WO_3_ particles and the amorphous carbon film of the TEM grid. This result remains hidden in the electrochemical current-potential data because the signal stems from the entire TEM grid electrode.

**Figure 4:**
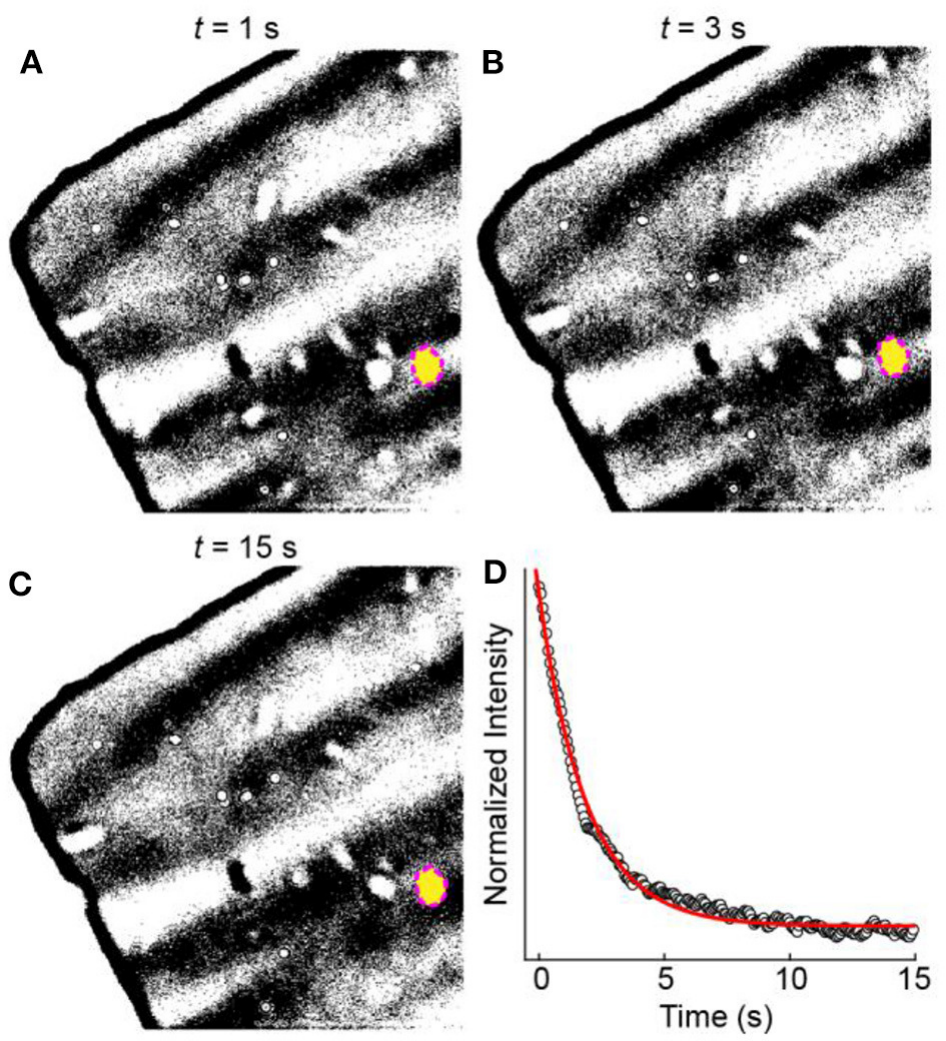
OD changes of the TEM membrane. **(A–C)** Optical microscope images as a function of time during a cathodic potential step experiment. **(D)** Normalized intensity vs. time trajectory (black circles) from the region of interest indicated by the yellow circle in **(A–C)**. The red line represents a fit to an exponential decay function.

To understand the origin of the large inactive population, we first examined the behavior of the amorphous carbon film substrate. We observed that bare regions of the amorphous carbon film exhibit an OD increase after the potential step even though no WO_3_ particles appear in that region (Figure 2e). To understand the role of the applied potential on the OD increase, we analyzed the OD dynamics of the amorphous carbon substrate. Figures 4A–C shows a sequence of bright field transmission images during the electro-optical imaging experiment. The image brightness and contrast were adjusted to highlight the small pixel intensity changes of the amorphous carbon film. Figure 4D shows a normalized intensity vs. time trajectory of the blank amorphous carbon film region (yellow oval in Figures 4A–C). The OD intensity changes immediately upon applying the cathodic potential pulse and decays exponentially with time. We attribute this exponential decay behavior to a mechanical response of the amorphous carbon film during the electrical double layer charging process of the membrane, which also occurs exponentially with time (Bard and Faulkner, 2001). The slow OD decay compared to electrical double layer charging is likely due to the slow mechanical motion of the film in the electrolyte. In this scenario, the OD increases because the membrane material folds into the region of space that is sampled in a single 222 × 222 nm^2^ pixel.

**Figure 5:**
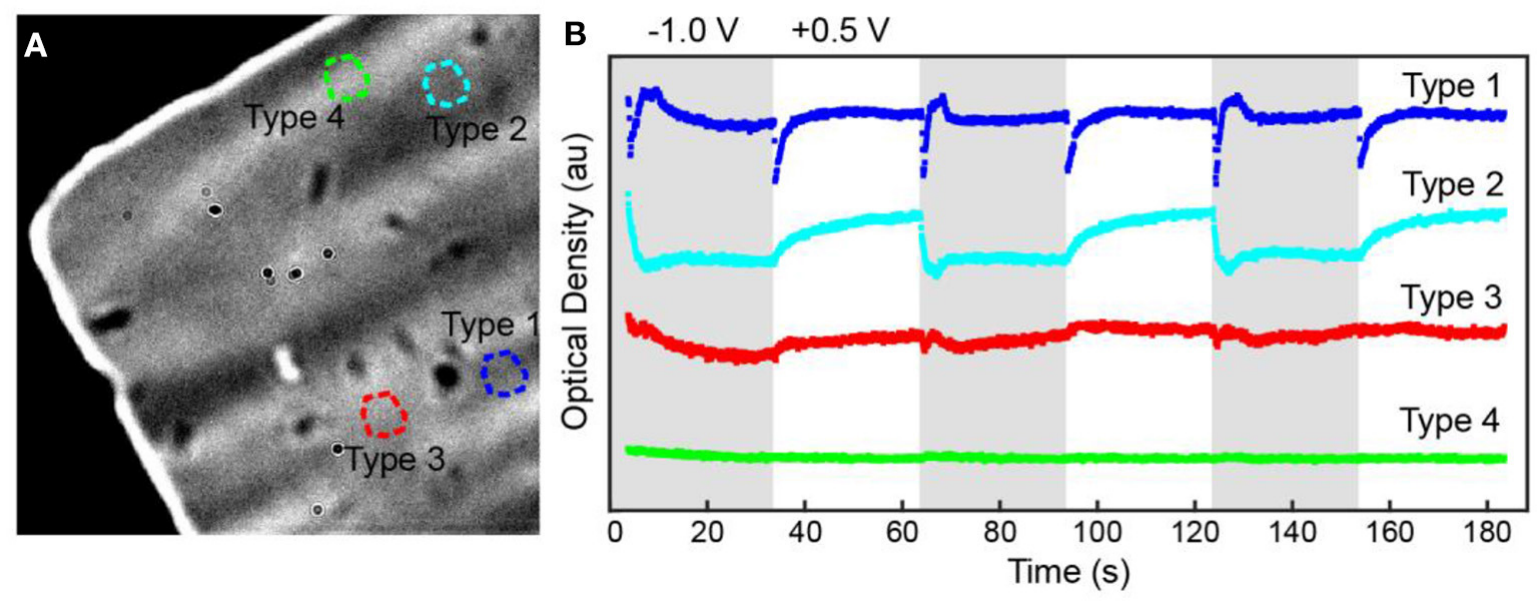
**(A)** Transmission image and **(B)** OD trajectories measured from four different substrate regions, labeled Type 1, 2, 3, and 4 in **(A)**.

We identified four types of mechanical behaviors after surveying 89 regions of the bare amorphous carbon film. Figures 5A,B shows four representative OD trajectories from different regions of the amorphous carbon-film. All regions show an overall OD decrease during the initial cathodic polarization pulse. This behavior suggests that the film stretches or bends, either during electrical double layer formation or upon lithiation of the amorphous carbon (Li and Wang, 2020), and then returns contracts to an initial state after cathodic polarization. After the initial pulse, Type 1 regions exhibit a sharp OD decrease upon applying a potential step, regardless of the polarization potential, followed by an OD increase during the cathodic polarization step. On the other hand, Type 2 regions exhibit a slow OD increase upon applying anodic potentials and a sharp OD decrease at cathodic polarization potentials. In this case, the electrode polarization influences the apparent thinning of the amorphous carbon material in the region of interest. That OD increases in one region and decreases in another suggests that the film could be stretching in some regions and bunching up in others. Type 3 regions exhibit slow, anomalous changes that can be linked to the polarization pulse whereas Type 4 regions show no optical changes, which could be due to either insulating areas of the amorphous carbon film or mechanically robust regions.

**Figure 6:**
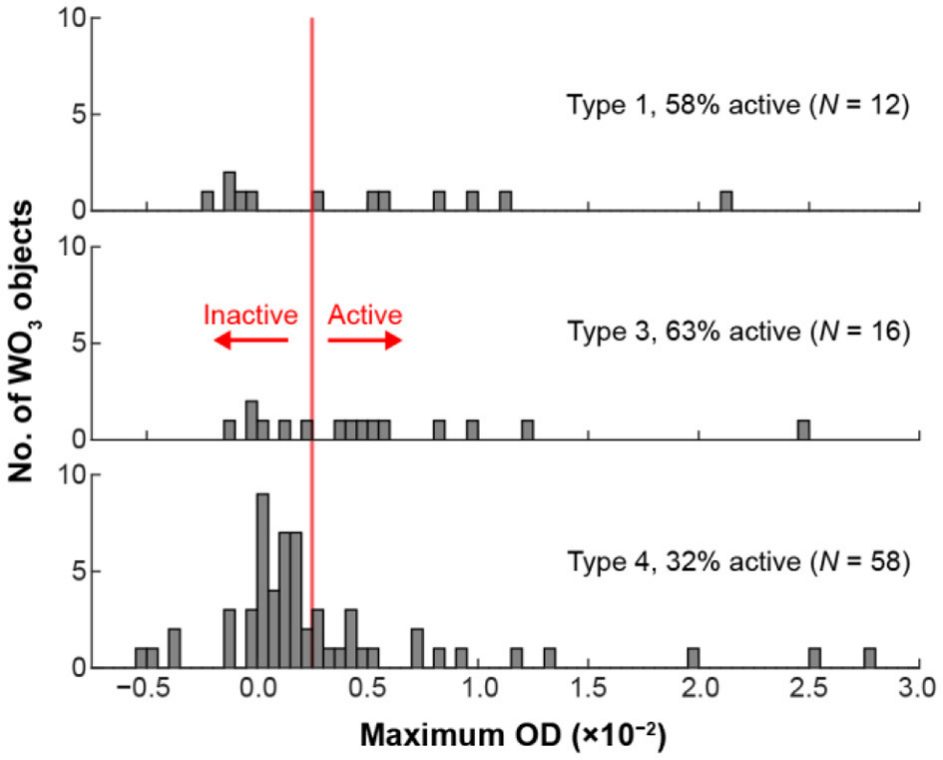
Distribution of maximum OD-values from 6 single WO_3_ NRs and 83 WO_3_ NR clusters consisting of 2–15 NRs located in different regions of the TEM grid. The TEM grid types were defined in Figure 5 (see main text for discussion).

We hypothesized that the electrochemical activity could be linked to the local behaviors of the underlying amorphous carbon film. To test this hypothesis, we examined the OD behavior of the amorphous carbon film surrounding each particle. Then, we assigned each h-WO_3_ NR to a population based on that local film behavior. Figure 6 shows the distribution of OD-values for 6 single WO_3_ NRs and 83 WO_3_ NR clusters for Type 1, 3, and 4 regions. We omit the three particles that were assigned to a Type 2 region because the results are not statistically meaningful, likely due to the fact that this type of mechanical behavior is not present across a large fraction of the TEM membrane material. The largest, but least active, particle population was WO_3_ particles in Type 4 areas. Here, we define active particles as those whose OD exceeds the average OD-value from Type 4 areas plus three standard deviations. The large inactive population could be due to the insulating nature of the TEM membrane material because we observed that Type 4 material did not respond to potential steps (Figure 5B). Less particles were observed in Type 1 and 3 areas, but those particles were, on average, more active. Type 1 and 3 areas are likely conductive regions because the material responds to the potential step (Figure 5B), which could explain the higher fraction of active particles in Type 1 and 3 regions.

Regardless of the underlying origin of the activity distributions in Figure 6, we conclude that the local mechanical properties of the TEM membrane influence the electrochemical activity of the h-WO_3_ NRs. It may be possible to test this hypothesis by making local force measurements using an atomic force microscope. Our results have broader implications for correlated electrochemical and *ex situ* TEM measurements that link ensemble-level electrochemical data with *ex situ* TEM imaging. The electrochemical current of TEM membrane-supported particles may not reflect the current on other current collectors and the current may vary from particle-to-particle due to support interactions.

**Figure 7:**
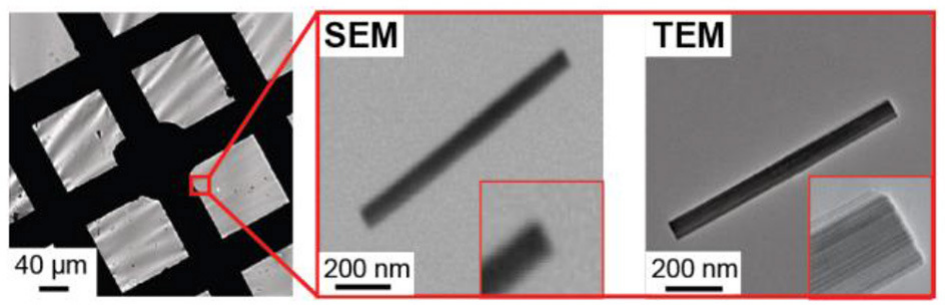
Comparison of SEM and TEM imaging results of the same single h-WO_3_ NR on the TEM grid working electrode.

An important aspect of this work that goes beyond our previous study is that correlated TEM imaging reveals atomic level structural detail that cannot be achieved with correlated SEM imaging, as is typically done in the field of single particle electrochemistry. Figure 7 compares SEM and TEM imaging of a single h-WO_3_ NR on the TEM grid working electrode. High resolution SEM imaging does not reveal atomic level structural features that appear in the TEM image. This capability will enable future studies that elucidate the role of intraparticle structural properties on single particle electrochemical behavior.

## Conclusions

We developed a correlated optically-detected electrochemistry/*ex situ* TEM imaging approach to study the electrochemical activity of single particles on a TEM grid electrode. The methodology presented herein can be applied generally to numerous electrochemical systems that exhibit optical property changes during electrochemical cycling (e.g., electrochromic smart windows, batteries, solid oxide fuel cells, and sensors). In the context of energy storage, the optical microscopy can be applied to study electrochromic materials such as LiCoO_2_ (Švegl et al., 2000), LiFePO_4_ (Zaghib et al., 2007), and Li_4_Ti_5_O_12_ (Yu et al., 2010; Li et al., 2019). The technique can be performed in reflectance mode on opaque current collectors such as Al, Ni, or Cu foil and is not limited to transparent current collectors. This analytical technique leverages a conventional bright field optical microscope to obtain single particle electrochemistry data and is compatible with existing TEM (Huang et al., 2010; Liu and Huang, 2011; Liu et al., 2012; Qi et al., 2016; Xie et al., 2017; Tu et al., 2018; Zhang et al., 2020) and X-ray (Totir et al., 1997; Ota et al., 2003; Deb et al., 2004; Kim and Chung, 2004; Chao et al., 2010; Shearing et al., 2011; Nelson et al., 2012, 2017; Shapiro et al., 2014; Wolf et al., 2017; Yau et al., 2017; Li et al., 2018; Yu et al., 2018) micro-spectroscopy methods.

## Data Availability Statement

The raw data supporting the conclusions of this article will be made available by the authors, without undue reservation.

## Author Contributions

CC and RE performed experiments, analyzed data, and wrote the manuscript. ZN performed TEM measurements. JS analyzed data and wrote the manuscript. All authors contributed to the article and approved the submitted version.

## Conflict of Interest

The authors declare that the research was conducted in the absence of any commercial or financial relationships that could be construed as a potential conflict of interest.
