# Supplementary material for: Local Substrate Heterogeneity Influences Electrochemical Activity of TEM Grid-Supported Battery Particles
Source: Front Chem. 2021 Mar 19;9:651248. doi: 10.3389/fchem.2021.651248 (PMC8017160; doi:10.3389/fchem.2021.651248)
Supplement: Supplementary file 1 [file Data_Sheet_1.PDF]

SUPPLEMENTAL INFORMATION FOR

Local substrate heterogeneity influences electrochemical activity of TEM grid-supported battery particles

Christina Cashen, R. Colby Evans, Zach N. Nilsson, Justin B. Sambur*

Colorado State University, Department of Chemistry, 200 W Lake St
Fort Collins, CO, 80523-1872

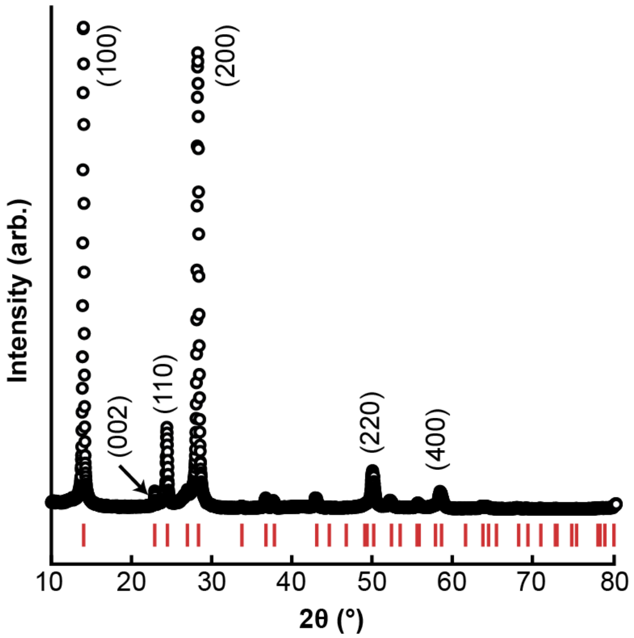

Figure S1. XRD pattern of hexagonal WO_3 sample (black data). The powder X-ray diffraction (PXRD) pattern of these samples matched that of a hexagonal WO3 (P6/mmm, JCPDS 00-033-1387JCPDS 01-081-0577, red lines).
